# Intraperitoneal Injection of Multiplacentas Pooled Cells Treatment on a Mouse Model with Aplastic Anemia

**DOI:** 10.1155/2016/3279793

**Published:** 2016-02-22

**Authors:** Jun Li, Hong Chen, Yan-Bo Lv, Qiang Wang, Zheng-Jun Xie, Li-Hua Ma, Jie He, Wei Xue, Shan Yu, Jun Guo, Ting-Hua Wang, Tian-Xi Wu, Xing-Hua Pan

**Affiliations:** ^1^Medical School, Kunming University, Kunming 650214, China; ^2^Stem Cell, Tissue and Organ Engineering Research Center, Kunming General Hospital of Chengdu Military Command, Kunming 650032, China; ^3^Department of Oncology, Kunming General Hospital of Chengdu Military Command, Kunming 650032, China; ^4^Department of Haematology and Endocrinology, Kunming General Hospital of Chengdu Military Command, Kunming 650032, China; ^5^Digestive System Department, Kunming General Hospital of Chengdu Military Command, Kunming 650032, China; ^6^Neuroscience Institute of Kunming Medical University, Kunming 650500, China

## Abstract

Coinfusion of hematopoietic and mesenchymal stem cells is more effective than hematopoietic stem cell transplantation alone. It is necessary to explore a safe and routine mixed stem cell intraperitoneal transplantation method. Multiplacentas pooled cells were intraperitoneally injected into a radiation- and immunity-induced mouse aplastic anemia model with single time. Then, mouse survival time, peripheral blood hemoglobin count, bone marrow architecture, and donor cell engraftment were assessed. The recipient mouse exhibited donor cell engraftment in both bone marrow and peripheral blood. Survival time and peripheral blood hemoglobin count increased in placenta pooled cells treated mice, compared with model-only controls (*P* = 0.048 and *P* = 0.000, resp.). However, placentas pooled cells failed to cause a significant decrease in bone marrow pimelosis area (*P* = 0.357). Intraperitoneally transplanted multiplacentas pooled cells can survive and engraft into a host body through blood circulation, which can increase the life span of an aplastic anemia model mice, and delay but not abrogate the development of aplastic anemia. Furthermore, they appear to play a role in increasing peripheral blood hemoglobin level response for increasing the life span of aplastic anemia model mice.

## 1. Introduction

Aplastic anemia is a refractory disease that has a high fatality rate, and the destruction of hematopoietic cells by the immune system leads to pancytopenia [1]. Stem cells exhibit promising treatment effectiveness [2]. However, it is currently not a routine clinical treatment. One possible reason is the different impacts of the sources of cells with different properties of cells in a given heterogeneous population on the same condition [3]. It is necessary to explore a new stem cell therapeutic measure.

Current cell therapy protocols utilize umbilical cord tissue derived mesenchymal stem cells as an alternative to bone marrow mesenchymal stem cells [4]. The placenta is often a clinical waste product. It contains plenty of more primitive and immature stem cells than the adult bone marrow and contains hematopoietic stem cells, umbilical cord derived mesenchymal stem cells, umbilical cord blood mesenchymal stem cells, placenta derived mesenchymal stem cells, and so on [5–16]. Thus, allogenic transplantation research has made use of these stem cells for their pluripotency and immunological properties [17–19].

It has been reported that the cotransplantation of mesenchymal and hematopoietic stem cells is safe and more effective than hematopoietic stem cell transplantation alone [20]. Kadekar et al. reported that placenta derived mesenchymal stem cells are the most suitable feeders for the* ex vivo* maintenance of functional hematopoietic stem cells [4]. In addition, we found that the coculture of multiunit umbilical cord blood mesenchymal stem cells can dramatically boost their proliferation (unpublished), which is in accordance with the idea that double-unit cord blood grafts improve engraftment and reduce relapse risk [21, 22]. Furthermore, several studies have shown that intraperitoneally transplanted stem cells could engraft into host multiorgans [23, 24]. Taken together, we explored the impact of intraperitoneal injection of multiplacentas deprived mixed cells treatment on a mouse model with aplastic anemia.

## 2. Materials and Methods

### 2.1. Mice

In order to induce an aplastic anemia model, two-month-old inbred female BALB/cBy (H2d) and DBA/2 (H2d) mice were obtained from Kunming Medical University and Google Organisms, respectively, and were bred and maintained in the SPF animal facility of Kunming General Hospital of Chengdu Military Command under standard care and nutrition. The local institutional review board of Kunming General Hospital of Chengdu Military Command, under the auspices of the National Ministry of Heath, approved all of experimental procedures used in this study. One hundred fifty recipient BALB/cBy mice were equally divided into two parts: Part 1 and Part 2, with a complete randomized design. Then, each part was equally divided into the model-only control (vehicle), the healthy normal control, and multiplacentas pooled cells treatment group. Each group contained 25 mice. Posttransplantation survival time was only observed in mice in Part 1, while other detections such as peripheral blood hemoglobin count, bone marrow architecture, and donor cell engraftment were performed in mice in Part 2.

### 2.2. Induction of Aplastic Anemia

BALB/cBy mice received a sublethal total body irradiation dose of 4 Gy from Model 143 ^137^Cesium *γ*-irradiator (JL Shepherd) one hour before lymph node cell infusion. Inguinal, brachial, and axillary infusion of lymph node cells were obtained from female DBA/2 mice, as previously described [25–27]; and they were infused into female BALB/cBy mice at 1 × 10^6^ cells per recipient to induce aplastic anemia.

### 2.3. Isolation of Placentas Cells

Allogene mice multiplacentas (containing placenta, umbilical cord, and umbilical cord blood) derived mixed cells were obtained from green fluorescent protein-expressing transgenic inbred parturient C57BL/6J mice (H2b, provided by the Neuroscience Institute of Kunming Medical University). Irregardless of gender, C57BL/6J F1 mice placentas were mixed together, grinded, and filtered through a 100-eye cell sieve mesh to obtain a single-cell suspension. Then, cells were centrifuged at 750 g for 20 minutes with gradient Percoll (GE Healthcare AB, Uppsala, Sweden; http://www.amersham.com/) to obtain a 1.070–1.090 g/cm^3^ cell density (rich in stem cell). Cells were washed twice in Nutrient Mixture F-12 (Gibco® DMEM/F-12) Media and counted using a Vi-Cell counter (Coulter Cooperation) [28]. Then, cells were ready for use.

### 2.4. Multiplacentas Pooled Cell Treatment

For multiplacentas pooled cells treatment, when bone marrow failure was already apparent at seven days after total body irradiation, 1 mL of mixed cell suspension in Nutrient Mixture F-12 Media was intraperitoneally injected into BALB/cBy mice in the multiplacentas pooled cells transplanted group at 1 × 10^7^ cells per mouse with single time. The model-only control mice received the same volume of vehicle, while healthy normal controls did not receive any treatment.

### 2.5. Survival Time, Peripheral Blood Hemoglobin Count, Bone Marrow Architecture, and Donor Cell Engraftment Detection

After transplantation, posttransplantation survival time of mice in Part 1 was examined and compared. When any mouse in Part 1 or Part 2 was almost dying, mice in Part 2 were initially bled for peripheral blood donor cell engraftment determination and hemoglobin count using an F-820 vet hematology analyzer (Sysmex, Japan). Then, mice were sacrificed to collect femurs for bone marrow donor cell engraftment and histologic examination.

Aplastic anemia concerned morphological changes of bone marrow architecture, which were observed by bone marrow histologic examination. The mouse left femur was fixed in 10% neutral buffered formalin, decalcified, embodied and sectioned into slides, and stained with hematoxylin and eosin. Slides were viewed using an Olympus microscope, and bone marrow morphology photographic images were captured at 400x magnification. The bone marrow pimelosis area per visual field was counted with a reticulum micrometer using the counting dots method of quantitative pathology.

Fluorescent protein tracing donor C57BL/6J mice cells engraftment and location were identified. The bone marrow was pushed out of the right femur of the mouse with the Nutrient Mixture F-12 Media and fully misced bene for antigrading. Each sample was smeared on the slide and subjected to fluorescence microscopy within a few hours. The image was acquired from the sample at 400x magnification and the number of green fluorescent protein positive cells per visual field was determined, as well as the peripheral blood smear.

### 2.6. Statistical Analysis

Summary statistics such as means and standard deviation were used to describe the mice samples' baseline characteristics. Group differences were examined by one-way ANOVA. For experiments that ANOVA justified, post hoc comparisons between group means were conducted using the LSD, S-N-K, and the Dunnett test for multiple comparisons. In the event in which only single experimental and control groups were used, group variance was examined by independent-samples *t*-test or the Wilcoxon Rank Sum Test. Differences were considered significant at *P* < 0.05. All analyses were performed using the IBM SPSS 18.0 software.

## 3. Results

All animals in Part 2 were bled and scarified when some mice were almost dying at day seven after transplantation for various analyses, as specified in each experiment.

### 3.1. Peripheral Blood Hemoglobin

**Figure 1:**
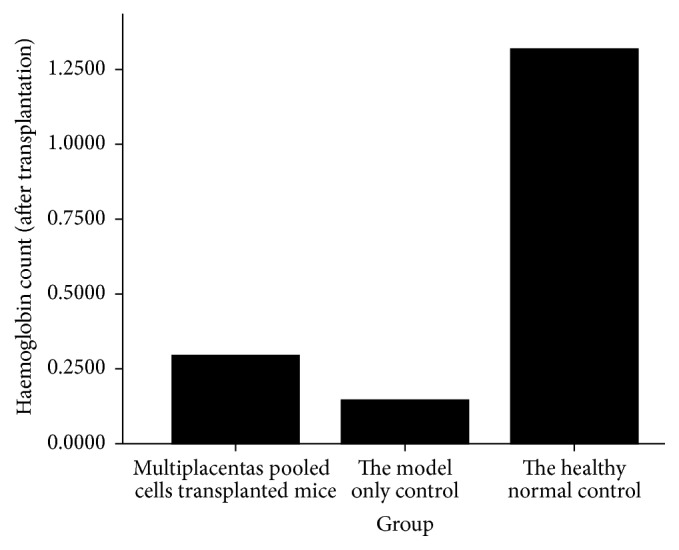
Mice peripheral blood hemoglobin count at day seven after placentas pooled cell treatment.

Peripheral blood hemoglobin count was performed automatically in a hematology analyzer. Hemoglobin count was notably higher in the placentas pooled cells treated groups than in the model-only control group (0.2948 ± 0.04629 versus 0.1460 ± 0.03808, *P* = 0.000). The number was as high as 1.3180 ± 0.03202 in healthy normal controls, which was significantly higher than model-only controls (*P* = 0.000, Figure 1).

### 3.2. Posttransplantation Survival Time

**Figure 2:**
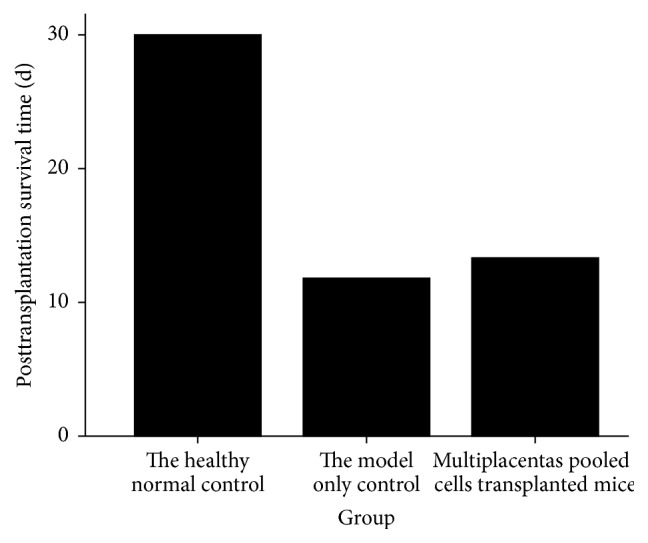
Posttransplantation survival time of mice in Part 1.

Mice in the healthy normal control group of Part 1 all survived until one month after transplantation. However, mortality to a 100% level was induced with aplastic anemia (other groups of Part 1) within 18 days. Thus, all mice in Part 1 were sacrificed at one month after treatment. Posttransplantation survival time in the healthy normal control group was significantly higher (*P* = 0.000) than in model-only controls. The survival time of the placentas pooled cells treated group (13.320 ± 2.704) was longer (*P* = 0.048) than in the model-only control group (11.800 ± 2.582, Figure 2).

### 3.3. Bone Marrow Histopathology

**Figure 3:**
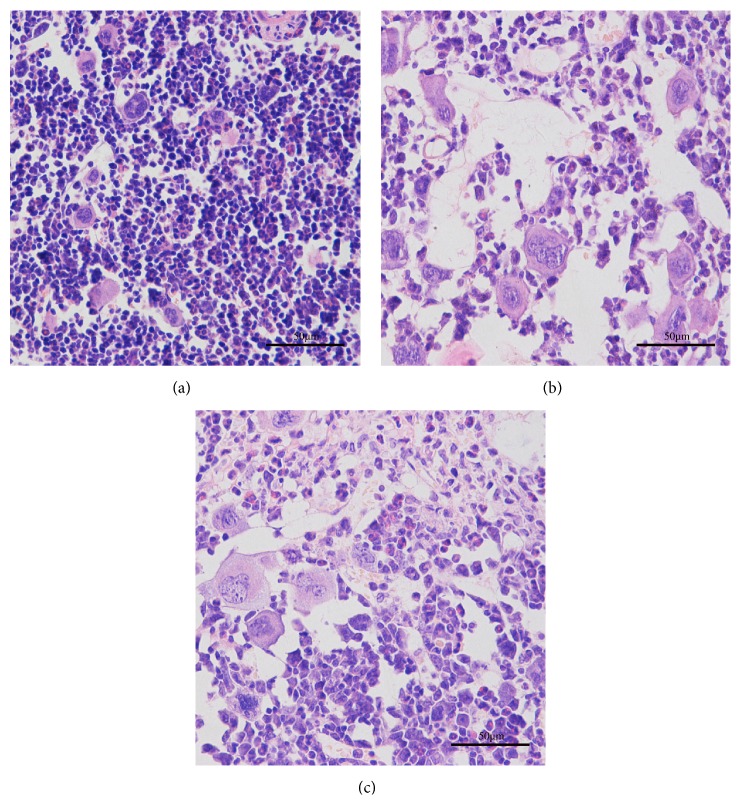
Mice bone marrow histopathology at day seven after placentas pooled cells treatment (400x). (a) Healthy normal controls; (b) model-only controls; (c) multiplacentas pooled cells transplanted mice.

Typical bone marrow histopathology was observed in both the normal and experimental groups. Hematoxylin and eosin staining of the bone marrow exhibited that multiplacentas pooled cells treatment in mice marrow, which is the same as model-only controls, were filled with adipocytes with large empty spaces, compared with healthy normal controls that exhibited a densely packed cellular distribution in normal condition (Figure 3).

### 3.4. Bone Marrow Pimelosis Area

**Figure 4:**
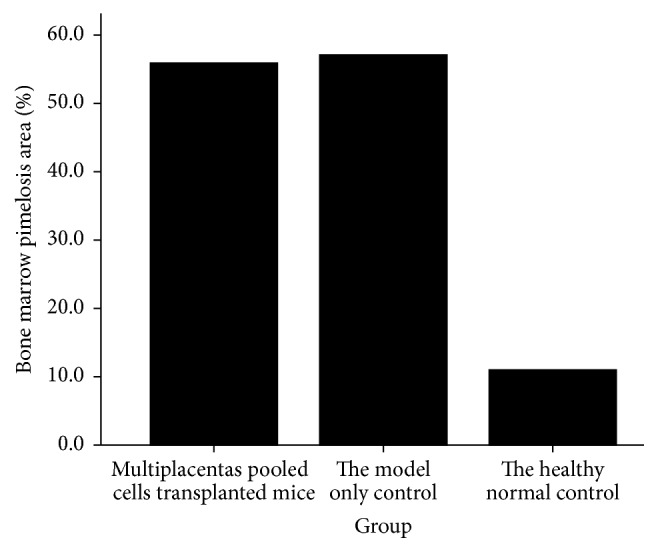
Mice bone marrow pimelosis area at day seven after placentas pooled cells treatment.

The bone marrow pimelosis areas in multiplacentas pooled cells treated mice, model-only controls, and healthy normal controls were 55.866 ± 6.0403%, 57.121 ± 3.8636%, and 11.031 ± 1.8310%, respectively. As shown in Figure 4, at day seven, the pimelosis area of healthy normal controls was significantly lower (*P* = 0.000) compared with model-only controls. At the same time, multiplacentas pooled cells treated mice had a lower pimelosis area than model-only controls, but the difference was not statistically significant (*P* = 0.357), as both groups had bone marrow hypocellularity.

### 3.5. Identification of Donor Cell

**Figure 5:**
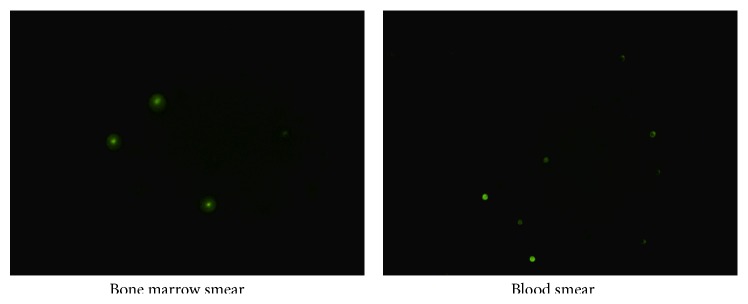
The multiplacentas pooled cells transplanted mice exhibited positive donor cell engraftment at day seven after transplantation (400x).

Twenty-five smear images were acquired for each group of mice in Part 2, and placentas pooled green fluorescent cells were observed. Every sample of mouse that received 10^7^ placentas pooled cells exhibited positive donor cell engraftment in both bone marrow and peripheral blood at day seven after transplantation (Figure 5), while other groups of mice in Part 2 did not reveal any positive cell (figure not shown).

## 4. Discussion

Our study assessed the therapeutic effect and cellular mechanism of placentas pooled cells on aplastic anemia and a safe, effective stem cell therapeutic measure. Placental mesenchymal stromal cells are very primitive and have multipotent differentiation potency. The expansion potency of placenta derived mesenchymal stromal cells is higher compared to the bone marrow [17, 29]. Placenta derived mesenchymal and hematopoietic stem cells are homologous [4]. All these may contribute to treatment effectiveness.

We administrated the latest extracted primary cells with single time in order to avoid immunologic facilitation induced by repeated administration. Peripheral blood hemoglobin count, survival time, and the bone marrow pimelosis area were compared between the model-only control group and healthy normal controls. All differences were statistically significant (all *P* = 0.000). The dates revealed that the radiation and immunity mediated model induced long-term aplastic anemia. Posttransplantation survival time dates indicate that the intraperitoneal transplantation of multiplacentas pooled cells can increase the life span of aplastic anemia model mice, and transplants are capable of delaying but not abrogating the development of aplastic anemia.

Treatment effectiveness depends on the number of transplanted cells. Currently, the number of patients transplanted with double umbilical cord blood has surpassed the number of receiving single cord blood unit [30]. Thus, we administrated 1 × 10^7^ multiplacentas pooled mixed cells per mouse with intraperitoneal injection transplantation. Mesenchymal stem cells can adhere to the peritoneum, which can support and carry it into the blood circulation [31]. In addition, we found the presence of donor cells in both the recipient's bone marrow and peripheral blood. It indicates that intraperitoneally injected placentas derived cells survive and engraft into the host body through blood circulation.

Multiplacentas pooled cells treatment mice had a lower pimelosis area than model-only controls, but the difference was not statistically significant. However, donor cells significantly increased in peripheral blood hemoglobin levels. Thus, placentas pooled cells appear to play a role in increasing peripheral blood hemoglobin level response for increasing the life span of aplastic anemia model mice. Pimelosis area decreased much lower than the level of increase in peripheral blood hemoglobin, which might explain why placentas pooled cells can only delay the development of aplastic anemia.

Treated cells contained plenty of hematopoietic stem cells, mesenchymal stem cells, hematopoietic progenitor cells, hemopoiesis precursor cells, erythrocytes, and leucocytes [5–16]. These cells can initially migrate into blood vessels and into the bone marrow of aplastic anemia. In particular, hematopoietic stem cells can differentiate into erythrocytes, leucocyte, and so on. In addition, mesenchymal stem cells can promote hemopoiesis and modulate immunoreaction induced by DBA/2 mouse lymphocytes injected into the host blood vascular system [32–35]. All these may contribute to its promising treatment effectiveness. However, although these stem cells may prolong survival time, they could not effectively improve the bone marrow pathology architecture of mice.

## 5. Conclusion

Results indicate that the intraperitoneal transplantation of multiplacentas pooled cells can increase the life span of aplastic anemia model mice and delay but not abrogate the development of aplastic anemia. Intraperitoneally transplanted cells can survive and engraft into the host body through blood circulation. Improvement of peripheral blood hemoglobin levels, but not bone marrow architecture response, probably explains the increase in survival time observed in this study.
